# Development of a Novel Collagen Wound Model To Simulate the Activity and Distribution of Antimicrobials in Soft Tissue during Diabetic Foot Infection

**DOI:** 10.1128/AAC.01064-16

**Published:** 2016-10-21

**Authors:** Bianca L. Price, Andrew M. Lovering, Frank L. Bowling, Curtis B. Dobson

**Affiliations:** a Medical Device Biology Group, Faculty of Biology, Medicine and Health, The University of Manchester, Manchester, United Kingdom; b Antimicrobial Reference Laboratory, Microbiology Department, Bristol, United Kingdom; c University of Manchester, Manchester Diabetes Centre, Manchester, United Kingdom

## Abstract

Diabetes has major implications for public health, with diabetic foot ulcers (DFUs) being responsible for significant morbidity and mortality. A key factor in the development of nonhealing ulcers is infection, which often leads to the development of biofilm, gangrene, and amputation. A novel approach to treating DFUs is the local release of antibiotics from calcium sulfate beads. We have developed a novel model system to study and compare the release and efficacy of antibiotics released locally, using collagen as a substrate for biofilm growth and incorporating serum to mimic the biochemical complexity of the wound environment. We found that our soft-tissue model supports the growth of a robust Pseudomonas aeruginosa biofilm, and that this was completely eradicated by the introduction of calcium sulfate beads loaded with tobramycin or gentamicin. The model also enabled us to measure the concentration of these antibiotics at different distances from the beads and in simulated wound fluid bathing the collagen matrix. We additionally found that a multidrug-resistant Staphylococcus aureus biofilm, nonsusceptible to antibiotics, nonetheless showed an almost 1-log drop in viable counts when exposed to calcium sulfate beads combined with antibiotics. Together, these data suggest that locally applied antibiotics combined with calcium sulfate provide surprising efficacy in diabetic foot infections and offer an effective alternative approach to infection management. Our study additionally establishes our new system as a biochemically and histologically relevant model that may be used to study the effectiveness of a range of therapies locally or systemically for infected DFUs.

## INTRODUCTION

Diabetes is an increasingly common disease, with 2.9 million people in the United Kingdom alone living with this condition, expected to increase to 5 million by 2025 (1). Around 15 to 25% of patients with diabetes develop diabetic foot ulcers (DFUs) in their lifetime (2, 3), and these have a multifactorial etiology, including alteration in foot architecture, peripheral neuropathy, and peripheral arterial disease (PAD), which put the foot at risk of tissue breakdown and subsequent ulceration (4). PAD is a key risk factor for limb loss due to diabetic foot infections (DFI) and has been identified in over half of all cases of DFIs in the United Kingdom (5). PAD leads to decreased nutrient exchange at the ulcer, causing hypoxia and ischemia (3). Autonomic dysfunction is also common in diabetic patients, which can cause skin to become dry and cracked, increasing the risk of developing infections (3). Patients with diabetes often do not exhibit a normal inflammatory response, indicating immunopathy (6).

Around half of DFUs become clinically infected, typically by opportunistic pathogens (7, 8). Infection is defined by a high load of pathogenic bacteria, evidenced by purulent discharge and local inflammation (9), and becomes chronic in around 10 to 15% of DFU cases (10, 11). Chronically infected DFUs are classified as nonhealing ulcers, characterized by prolonged inflammation and progressive tissue damage with biofilm involvement (12). Such ulcers typically take more than 2 months to heal and a third will require surgery (11, 13, 14).

Recommended management of DFIs includes surgical debridement and taking cultures from debrided tissue for microbiological examination, accompanied by systemic treatment with antibiotics (15, 16). Antibiotics are administered orally or intravenously depending on the severity of infection. Broad-spectrum antibiotics are used initially and the selection is later adjusted according to the clinical and microbiological response (1).

For patients with chronic wounds that are poorly vascularized, such as those with PAD, and which have persistent infections with biofilm involvement, topical antimicrobial therapy may be a useful alternative to systemic antibiotics (17) for clearing infectious organisms from complex wounds. There is a wide variety of topical antimicrobials, including silver dressings, chlorhexidine, honey, and iodine, and also antibiotics applied as a powder or formulated into solutions, creams, and ointments.

Calcium sulfate (Stimulan; Biocomposites, Ltd.) has been approved for use as a bone void filler which is absorbed *in vivo*. The material has the ability to be combined with antibiotics and molded into beads. Antibiotics released from the beads have been shown to be efficacious in osteomyelitis in a rabbit model (18). A study of 354 osteomyelitis patients who had intact local perfusion and in whom previous or ongoing treatment had failed were treated with vancomycin and gentamicin combined with calcium sulfate beads. This study reported that 86.4% of patients clinically healed without use of intravenous antibiotics (19). Calcium sulfate has also been used with gentamicin and vancomycin in case studies of DFI (20, 21).

Although simple elution studies have established that antibiotics are steadily released from calcium sulfate beads when placed in a liquid environment, the clinical setting of a wound bed infected with a biofilm is complex, and both elution and efficacy may be influenced by the presence of biofilm or diverse host biomolecules. Therefore, we have adapted a soft-tissue wound model of an established biofilm that was previously shown to effectively mimic bacterial aggregates in a chronic wound infected with P. aeruginosa (22). This closed culture system utilizes type 1 collagen as a substrate for bacterial attachment, because this is the primary proteinaceous component of the dermis and additionally utilizes simulated wound fluid (SWF), as established previously (22, 23).

Our new model features a void into which can be introduced a clinically relevant quantity of calcium sulfate beads. Here, we report the use of the model to compare the movement of different eluted antibiotics through the model soft-tissue collagen matrix and assess the antibiotic susceptibility of an established biofilm formed by susceptible or multidrug-resistant organisms. We first examined P. aeruginosa, a common and devastating DFU pathogen (24, 25) shown in a previous study to form a clinically relevant biofilm under similar conditions (22), and extended the study to include multidrug-resistant S. aureus (MDRSA). The latter is an especially challenging organism to treat with antibiotics, and when grown as a biofilm *in vitro*, it was insusceptible to the antibiotics used in the study. We used this organism to test whether the biofilm in the collagen model shows the same insusceptibility to these antibiotics.

## MATERIALS AND METHODS

### Bacterial strains, media, and culture conditions.

The Nottingham wild-type Pseudomonas aeruginosa PAO1 strain was used; PAO1-N was kindly donated by Miguel Camara (University of Nottingham). The multidrug-resistant strain Staphylococcus aureus subspecies aureus Rosenbach (ATCC 4330) (MDRSA) was purchased from LGC Standards (Teddington, United Kingdom). Overnight cultures were prepared in tryptic soya broth (TSB) (Oxoid) at 37°C and diluted 1/10,000 to 10^5^ cells/ml in simulated wound fluid (SWF). SWF is composed of 50% fetal bovine serum (FBS) (Sigma) and 50% peptone water (PW), which is 0.9% NaCl in 0.1% peptone. We used 500 μl of this cell suspension to inoculate the collagen wound model. Cultures were diluted to 10^5^ cells/ml in TSB for MIC assays or in SWF for minimum biofilm eradication concentration (MBEC) assays. Cell suspensions reached an optical density at 600 nm (OD_600_) of 2.5 to 3 when incubated in TSB; however, the OD_600_ in SWF was 1.5 at stationary phase. Biofilm formation in the MBEC assay was also increased in SWF, as expected in this minimal medium.

### Preparation of collagen wound models.

**FIG 1:**
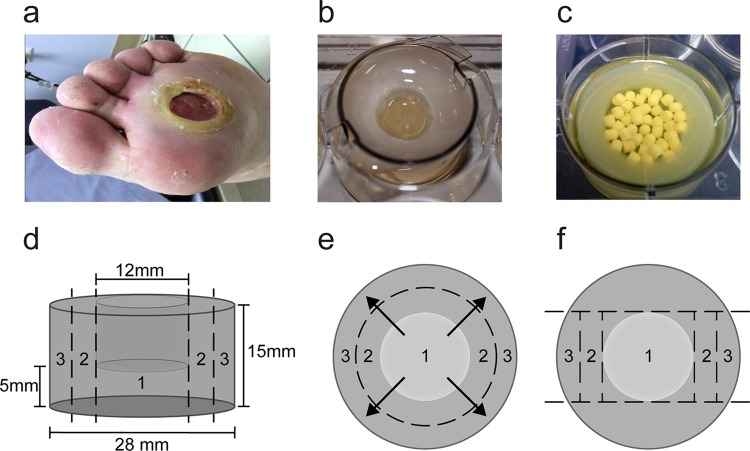
Collagen wound model is designed to represent a grade 1B diabetic foot ulcer. (a) An example of a grade 1B ulcer on the University of Texas scale, which denotes a superficial soft-tissue wound with infection. (b) The model was designed to represent a grade 1B ulcer. The collagen matrix was situated in a tissue culture well insert in a 6-well plate that was bathed in media. A void was created in the model using a mold. (c) Appearance of a model with a 20-mm void after incubation with PAO1 containing 1.25 g loaded calcium sulfate beads. (d) The model utilized for all experiments has a void that is 12 mm in diameter and 10 mm deep. (e) Calcium sulfate beads (unloaded) or combined with antibiotic (loaded) were placed into the void of the model, and the movement of antibiotics from the void to the collagen matrix beneath the void (area 1), the collagen adjacent to the void (area 2), or the edge of the model (area 3) and the medium after 3 or 7 days of incubation was determined. (f) Following incubation of the model with loaded or unloaded calcium sulfate beads, the model was sectioned. Colony counts and concentrations of antibiotics were determined for each section.

We used a closed-system, high-exudate model to quantify the antibiotic released over 3 days and considered DFIs that had reduced drainage. The Lavery et al. model was scaled up to represent a grade 1B ulcer (26) (Fig. 1a), using either a 6-well plate or, for all experiments in the current study, a transwell insert (3-μm pore size; HD PET track-etched membrane; Falcon) to which SWF was added (Fig. 1b). A schematic of the model in a 6-well plate shows its dimensions (Fig. 1d), and the movement of antibiotic from the void in area 1, where the beads are placed, through to area 2, adjacent to the void, and to area 3, at the edge of the model, was measured (Fig. 1e). For 10 ml collagen solution, 2 ml of 10 mg/ml type 1 collagen from rat tail (BD) was combined with 6 ml cold SWF, 1 ml 0.1% acetic acid, and 1 ml 0.1 M NaOH, and the final pH was adjusted to 7.5. We pipetted 8 ml collagen solution into each insert and placed a mold over the 6-well plate to create a void in each well as the collagen polymerized at 37°C for 1 h; void diameter could be varied from 12 mm to 20 mm for 28-mm inserts, with 12-mm voids being utilized for all experiments, as this was comparable to the size of a grade 1B ulcer (6 mm to 40 mm), which we had set out to model (27). After polymerization, 6 ml SWF was added to both the well and insert. The models were inoculated with PAO1 or MDRSA as appropriate before incubation at 37°C for 24 or 72 h.

### Exposure of collagen wound models to calcium sulfate beads.

A 10-ml pack of Stimulan (Biocomposites, Ltd., United Kingdom) contains 20 g of pharmaceutical-grade calcium sulfate alpha-hemihydrate powder and 6 ml of water. For the purposes of this study, 20 g of calcium sulfate was combined with antibiotic powder or solution or with sterile water (unloaded control). For gentamicin and tobramycin, three 2-ml vials of clinical-grade 40 mg/ml gentamicin or tobramycin were combined with 20 g calcium sulfate to make the 100% loaded beads; the final concentration was 240 mg antibiotics per 10 ml of calcium sulfate, as used clinically (19). To make beads at 50%, 25%, and 12.5%, the clinically used concentration, the appropriate amount of 40 mg/ml solution of gentamicin or tobramycin was added to 20 g calcium sulfate, and the final liquid volume was adjusted to 6 ml using sterile water. For calcium sulfate beads combined with gentamicin and vancomycin, 1 g of powdered vancomycin and three 2-ml vials of 40 mg/ml gentamicin were added to 20 g calcium sulfate. Resultant paste was cured in a mold to create 3-mm-diameter hemispherical beads (provided by Biocomposites, Ltd., United Kingdom). The void created by the mold in the collagen could hold up to 1.25 g of beads, which is approximately 50 beads (Fig. 1b and c). Beads were incubated in each model at 37°C for 24 h, 72 h, or 7 days.

### Quantitation of bacteria and antibiotics in collagen matrices.

Following incubation, the collagen wound model was removed and sectioned as shown in Fig. 1f. The SWF was removed from the system and the volume recorded. A 500-μl aliquot of SWF medium was taken and bacteria were pelleted. The supernatants were retained on ice for antibiotic concentration determination (see below). The bacterial cell pellet was resuspended in 500 μl phosphate-buffered saline (PBS) (Roche) and serially diluted to 10^−8^ before plating for colony counts. The collagen phase of the model was removed, weighed, and sectioned using a scalpel. One volume of collagenase solution (500 μg/ml in PBS; MP Biomedicals) was added to each section of the model. Collagen was dissolved over 90 min at 37°C, with vortexing every 30 min (22). Cells were pelleted by centrifugation at 13,000 × *g* for 3 min, and the supernatants were stored on ice. The cells were washed twice as stated above; wells containing loaded and unloaded beads were treated in the same way. The pellet was resuspended in one volume of PW, serially diluted to 10^−8^, and plated for colony counts. Supernatants from both the medium and collagen phases of the model were assessed for antibiotic concentration. Gentamicin, tobramycin, and vancomycin were assayed using an Olympus AU400 analyzer with the following commercially available kits: Cedia for gentamicin (Thermo Scientific Ltd.), QMS for vancomycin (Thermo Scientific Ltd.), and Emit 2000 for tobramycin (Beckman Ltd.). The kits were used and validated as detailed by the manufacturers' instructions with the following lower limits of quantitation: gentamicin, 0.3 μg/ml; tobramycin, 0.5 μg/ml; and vancomycin, 2.5 μg/ml. For all three assays, intra-assay precision (as assessed by the coefficient of variation of quality-control samples run at the same time) was less than 7%, while interassay precision was less than 10%. Samples with concentrations above the calibration range of the assay were diluted in phosphate-buffered saline (gentamicin and tobramycin) or normal human serum (vancomycin) with the results corrected for any dilutions. All *P* values were determined using a paired Student *t* test, and experiments were carried out in triplicate.

### Histological analysis.

Collagen wound models were inoculated with PAO1 and incubated for 24 h as described above but without a void molded into the collagen. An 8-mm cork borer was used to harvest samples for histology, which were snap-frozen. Frozen samples were sectioned to 20 μm in a cryostat. Samples were collected on X-tra adhesive slides (Leica) and stained using hematoxylin-van Gieson (HVG), hematoxylin-periodic acid-Schiff (PASH) (24), and 4′,6-diamidino-2-phenylindole (DAPI) (Invitrogen) by following the manufacturer's instructions.

### SEM.

Collagen wound models inoculated with PAO1 and incubated for 72 h were sampled as described for the histological analysis using a cork borer and fixed in 2.5% glutaraldehyde and 4% paraformaldehyde in 0.1 M HEPES (Sigma) for 1 h. These were washed five times for 5 min in 0.1 M HEPES, stained in 1% OsO_4_ in 0.1 M HEPES for 1 h, and then washed for 5 min four times in distilled H_2_O. Samples were dehydrated in ethanol and then critical point dried and sputter coated with gold-palladium before examination using an FEI Quanta 250FEG scanning electron microscope (SEM).

### MIC and MBEC assays.

MIC assays were carried out according to the CLSI guidelines (28). In brief, a dilution series was produced from a stock solution of 5 mg/ml gentamicin or tobramycin. For MICs with an MDRSA strain, vancomycin was combined with gentamicin (Sigma) at a ratio of 1:0.24, and stocks of 5 mg/ml vancomycin and 1.2 mg/ml gentamicin were made. The stock antibiotic concentration (1,024 μg/ml) was diluted 1:1 with bacterial overnight culture and sequentially diluted in 96-well plates to produce final antibiotic concentrations between 1 μg/ml and 512 μg/ml in 200 μl. The OD_600_ was read after overnight incubation. MBEC assays were adapted from the Innovatech protocol to match culture conditions used in our wound model. In brief, 200 μl of overnight culture diluted into SWF as described above was inoculated into each well of a 96-well plate. A peg lid was placed onto the 96-well plate and incubated for 72 h at 37°C. After a brief wash, pegs were incubated in antibiotics at 37°C for 72 h and then washed in PBS before incubation in fresh TSB overnight at 37°C, followed by measuring the OD_600_.

## RESULTS

### Assessment of biofilm growth in the collagen matrix.

**FIG 2:**
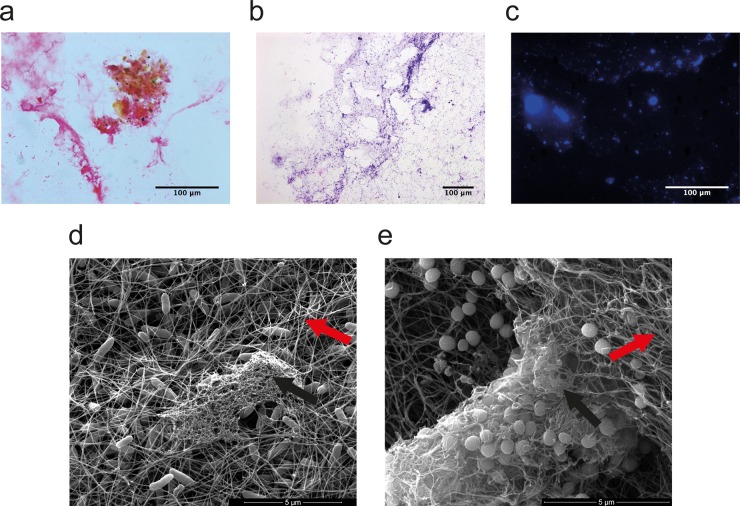
Characterization of biofilm formation in the collagen matrix. P. aeruginosa was incubated in the collagen wound model for 24 h, and then the model was sectioned and histologically stained. (a) HVG staining shows the collagen matrix (pink) as well as the presence of a cluster of bacterial DNA (stained black) showing bacterial cells growing in a microcolony. (b) PASH staining shows the presence of polysaccharide extracellular matrix (purple) surrounding bacterial DNA (stained dark blue). (c) DAPI fluorescent stain shows clusters of bacterial DNA in microcolonies within the collagen matrix. P. aeruginosa (d) and S. aureus (e) then were incubated in the collagen matrix for 72 h, and sections were imaged by SEM. Rod-shaped P. aeruginosa (d) and S. aureus (e) cocci can clearly be seen in each model. Polymerized collagen fibrils indicated by red arrows form a mesh that the bacteria grow in and interact with. Evidence of extracellular matrix produced by the bacteria is indicated with black arrows.

We prepared collagen wound models in 6-well plates inoculated with either S. aureus or P. aeruginosa (Fig. 1), and after 24 h of incubation we investigated whether the matrix showed characteristics of biofilm physiology. HVG staining of sections of the collagen showed bacterial DNA (black) within the collagen matrix (pink) (Fig. 2a). PASH was used to stain bacterial DNA (black) and confirmed the presence of a polysaccharide matrix (purple) (Fig. 2b). The fluorescent DNA stain DAPI enabled visualization of microcolonies within the section (Fig. 2c), suggesting the development of biofilm. An additional collagen wound model confirmed the presence of bacterial biofilm encased in an extracellular matrix and enabled the interactions of individual microbes and microcolonies associated with polymerized collagen fibers to be visualized (Fig. 2d and e).

### Efficacy of tobramycin-loaded beads against a P. aeruginosa biofilm and assessment of release of tobramycin into the collagen matrix.

**FIG 3:**
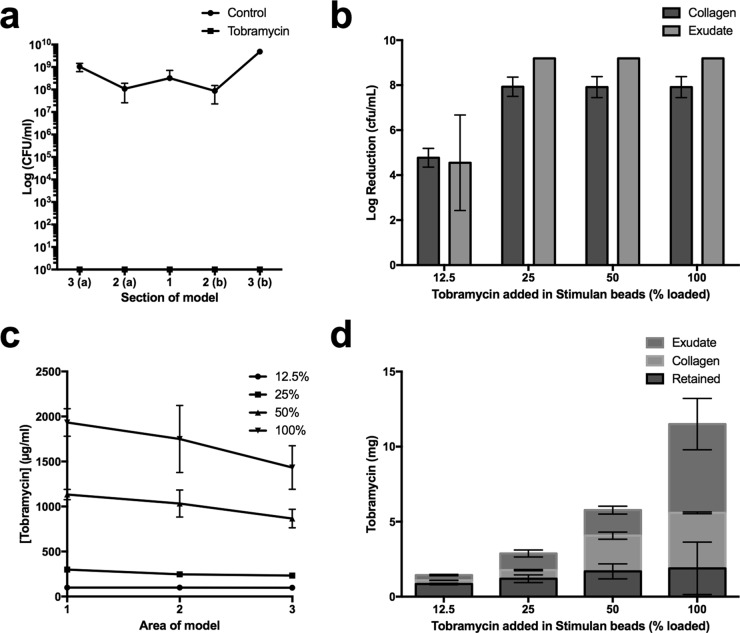
Growth inhibition of P. aeruginosa biofilm by tobramycin-loaded calcium sulfate beads. (a) Colony counts from a collagen wound model incubated with P. aeruginosa for 24 h and then calcium sulfate beads for 72 h. Colony counts are plotted for each area of the model; (a) and (b) on the axis label denote sections from each side of the central void. Circles show bacterial growth in the presence of control beads not loaded with antibiotic. Squares show colony counts for models that were incubated with 100% tobramycin-loaded beads; no viable counts were recovered. (b) Log reductions in bacterial cell counts after 72 h of incubation of P. aeruginosa in the model followed by a further 72 h of incubation with calcium sulfate beads. Data show the decrease in viable organisms after exposure to tobramycin relative to numbers of viable organisms on exposure to unloaded control beads. (c) Concentration of tobramycin in area 1 of the model underneath the void, to which beads are added, through to area 3 at the edge of the model. (d) Mass of tobramycin detected in the collagen and medium phase of the model (tobramycin not detected is presumed to be retained in the beads).

A biofilm formed by the opportunistic pathogen P. aeruginosa PAO1 was allowed to develop in the collagen wound model over 24 h. Beads loaded with tobramycin at levels previously reported clinically (100% loaded) or unloaded control calcium sulfate beads were placed in the model void (19). Wound models were incubated for a further 72 h before sectioning and digesting the collagen to enable viable counts to be determined. No bacterial counts were obtained for models incubated with calcium sulfate beads combined with tobramycin; however, counts of 10^8^ CFU/ml were present in collagen exposed to unloaded beads (Fig. 3a).

Due to the high efficacy of 100% tobramycin-loaded beads against the 24-h established biofilm, we tested the extent of release of tobramycin into the wound model, and its corresponding antimicrobial effect was measured on a more mature P. aeruginosa biofilm, established over 72 h. We expect the amount of biofilm in the model to increase with a longer incubation time (25); therefore, we increased the challenge for the antibiotics eluted from the beads. We tested beads loaded with 100%, 50%, 25%, and 12.5% of the clinically reported concentration (CRC) of tobramycin to assess the minimum amount necessary to eradicate the biofilm. After adding beads, models were incubated for a further 72 h. Application of beads loaded with 25% or more of the CRC of tobramycin eradicated the biofilm. Beads loaded with 12.5% of the CRC caused a 4.8-log reduction in viable counts (Fig. 3b).

For the 100% and 50% loaded beads, the highest concentration of tobramycin was found to be in the center of the model, adjacent to the wound void, with progressively lower concentrations toward the edge of the model (Fig. 3c), and concentrations of tobramycin found in the media were not significantly different from those in the collagen (*P* > 0.05). Tobramycin concentrations in the medium for the 25% loaded beads (470 ± 80 μg/ml) and the 12.5% loaded beads (170 ± 10 μg/ml) were significantly higher than concentrations measured in collagen (250 ± 50 μg/ml and 100 ± 10 μg/ml, respectively) (*P* < 0.01) (Fig. 3c), suggesting tobramycin does not associate strongly with the collagen matrix. On average, 41% (±3%) of the tobramycin originally added to the model in the calcium sulfate beads was detected after 72 h of incubation in the collagen or the medium for the 12.5% loaded beads, with the proportion detected increasing to 84% (±15%) for the 100% loaded beads (Fig. 3d). The remainder of the antibiotic is presumably retained within the beads.

### Efficacy of gentamicin-loaded beads against a P. aeruginosa biofilm and assessment of gentamicin release into the collagen matrix.

**FIG 4:**
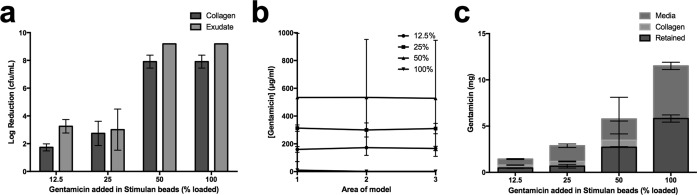
Growth inhibition of P. aeruginosa by gentamicin-loaded calcium sulfate beads. (a) Log reductions in bacterial cell counts after 72 h of incubation of P. aeruginosa in the model followed by a further 72 h of incubation with calcium sulfate beads. Data show decreases in viable organisms after exposure to gentamicin relative to numbers of viable organisms on exposure to unloaded control beads. (b) Corresponding concentration of gentamicin in area 1 of the model underneath the void, to which beads are added, through to area 3 at the edge of the model. (c) Mass of gentamicin detected in the collagen and medium phase of the model (gentamicin not detected is presumed retained in the beads).

We applied calcium sulfate beads loaded with 100% of the gentamicin CRC to a mature P. aeruginosa biofilm that had developed in the collagen wound model over 72 h. Beads loaded with 50%, 25%, and 12.5% of the CRC of gentamicin were also tested in the model. After incubation of beads in the models for a further 72 h, no viable counts for models treated with 100% or 50% loaded beads were recovered. Application of 25% and 12.5% loaded beads caused 2.7- and 1.7-log reductions in viable counts, respectively (Fig. 4a).

Gentamicin concentrations were homogenous throughout the collagen matrix (Fig. 4b). Concentrations of gentamicin in the media were 1.5- and 1.6-fold higher than those in the collagen for models to which 12.5% or 25% CRC beads were added (*P* > 0.001) (Fig. 4c). The concentration of gentamicin was the same in the medium and the collagen in models to which the 50% beads were added (460 and 530 μg/ml in medium and collagen, respectively) (*P* < 0.001), but in the model to which 100% loaded beads were added, almost all of the gentamicin was detected in the medium (2,300 and 4 μg/ml in the medium and collagen, respectively).

The amount of gentamicin detected in the medium and the collagen combined was variable. On average, 49% (±3%) of the gentamicin loaded into the 100% beads was detected in the medium or collagen. The amount recovered was 53% (±49%) for 50% loaded beads, 77% (±7%) for 25% loaded beads, and 68% (±3%) for 12.5% loaded beads. In all cases the remainder is presumably retained within the beads.

### Efficacy of gentamicin- and vancomycin-loaded beads against a multidrug-resistant S. aureus biofilm and assessment of antibiotic release into the collagen matrix.

**FIG 5:**
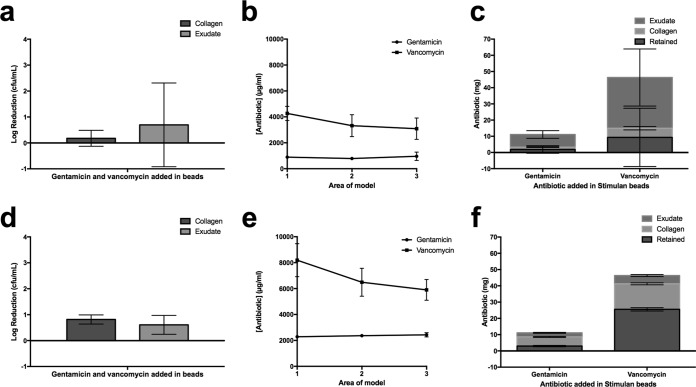
Growth inhibition of MDRSA after incubation with vancomycin- and gentamicin-loaded calcium sulfate beads for 72 h and 7 days. (a) Log reductions in bacterial cell counts after 72 h of incubation of MDRSA in the model followed by a further 72 h of incubation with calcium sulfate beads loaded with vancomycin and gentamicin. Data show the decrease in viable organisms after exposure to antibiotics relative to numbers of viable organisms on exposure to unloaded control beads. (b) Corresponding concentration of antibiotics in area 1 of the models shown in panel a underneath the void, to which beads are added, through to area 3 at the edge of the model. (c) Mass of antibiotics detected in the collagen and medium phase of the models shown in panel a. (d) Log reductions in bacterial cell counts after 72 h of incubation of MDRSA in the model followed by a further 7 days of incubation with calcium sulfate beads loaded with vancomycin and gentamicin. Counts from models exposed to antibiotics are subtracted from counts for unloaded control beads. (e) Corresponding concentration of antibiotics in area 1 of the models shown in panel d underneath the void, to which beads are added, through to area 3 at the edge of the model. (f) Mass of antibiotics detected in the collagen and medium phase of the models shown in panel d.

To further establish the ability of our model to reflect the antibiotic susceptibility of diverse wound flora, we examined the efficacy of gentamicin- and vancomycin-loaded beads on an MDRSA strain. We selected a methicillin-resistant S. aureus strain (ATCC 43300) which we had found to be resistant to gentamicin (MIC, 512 μg/ml) (Table 1) and which showed resistance to vancomycin when growing as a biofilm in SWF (MIC, 2 μg/ml; MBEC, 512 μg/ml) (Table 1). A mature MDRSA biofilm was established over 72 h prior to introduction of 100% loaded beads and incubation for a further 72 h. For this strain, we measured a 0.18-log reduction in CFU in the collagen phase of the model (Fig. 5a).

**TABLE 1:** MBC, MIC, and MBEC data for P. aeruginosa and S. aureus^*a*^

Strain and antibiotic	Growth data (μg/ml)		
	Planktonic MIC	Biofilm^*b*^	
		MBEC	Model MBC
P. aeruginosa PA01			
Tobramycin	1	8	252
Gentamicin	8	31.3	532
MRSA ATCC 6538			
Gentamicin	512	>512	>2,500
Vancomycin	2	512	>2,100
Gentamicin-vancomycin	0.12/0.5	16/66.7	>2,400/6,590

a MIC and MBEC data for P. aeruginosa and S. aureus were compared to MBC data for local concentrations of antibiotics determined in the collagen phase of the wound model.

b Where “>” is used, the highest concentration of antibiotics used in the MBEC or detected in the collagen is cited but bacterial growth was still present. Each assay was set up with *n* = 8 and repeated on two different days.

Concentrations of vancomycin released from the beads were 4,300 μg/ml in section 1 of the model, and this decreased to 3,100 μg/ml in section 3 (*P* < 0.05). The corresponding gentamicin concentrations were, on average, 900 μg/ml in the collagen with no detectable concentration gradient from central area 1 of the model (Fig. 1d).

On average, 83% (±21%) of the gentamicin and 80% (±39%) of the vancomycin combined with calcium sulfate beads was recovered from the medium and collagen phase of the model (Fig. 5c).

These experiments were repeated again with a 72-h biofilm, but beads were incubated in the model for 7 days rather than 72 h. Here, larger log reductions of 0.82 were observed (Fig. 5d). Increased concentrations of vancomycin and gentamicin in the collagen phase of the model were observed. In area 1 of the model, 8,200 μg/ml vancomycin was measured, decreasing to 5,900 μg/ml in area 3 (Fig. 1d), although this difference was not significant. Gentamicin concentrations were again homogenous throughout the collagen phase of the model but were higher than those observed in the 72-h model at 2,400 μg/ml (Fig. 5e). Consistent with previous data, more vancomycin and gentamicin was measured in the medium phase of the model than the average concentration in the collagen phase (6,800 and 3,300 μg/ml, respectively).

### Planktonic and biofilm MICs and MBECs.

To enable us to compare the efficacy of antibiotics in the new collagen wound model with that for the antibiotics in standard laboratory assays, MIC and MBEC assays were carried out. As expected, for both P. aeruginosa and MDRSA, the measured MBEC, which was carried out for 72 h in SWF minimal medium, was higher than the MIC, and it was considerably so for MDRSA with vancomycin, where the MIC was 2 μg/ml and the MBEC was 512 μg/ml.

We additionally established minimum bactericidal concentrations (MBCs) for the antibiotics in the wound model. For the P. aeruginosa biofilm in the collagen wound model, the concentration of antibiotics required to eradicate the biofilm was 252 and 532 μg/ml for tobramycin and gentamicin, respectively, compared to 8 and 31 μg/ml for the MBEC (Table 1). The combination of gentamicin and vancomycin against S. aureus lowered the concentrations required for the MIC and MBEC; however, the highest concentrations achieved in the wound model were unable to eradicate the established MDRSA biofilm, although a 0.8-log reduction was achieved.

## DISCUSSION

The new soft-tissue wound model we have developed enables the release and efficacy of locally placed antibiotics to be measured and compared under conditions more relevant to the clinic than current biofilm models (23). Our use of SWF in the model results in high exudate conditions likely to present challenging conditions for antibiotic efficacy. Serum protein binding is known to be an important factor in antibiotic efficacy *in vivo* (26), and *in vitro* biofilm models with growth established for 72 h under high exudate conditions are difficult to eradicate with silver (23).

Calcium sulfate that had been combined with 240 mg of gentamicin or tobramycin as reported clinically (100% loaded) was added to the wound model as beads, resulting in 11,500 μg of each antibiotic placed into the model void (Fig. 1c), and this was sufficient to eradicate the P. aeruginosa biofilm growing in the collagen matrix. Tobramycin released from calcium sulfate beads had greater efficacy than gentamicin in the collagen phase of the wound model, completely eradicating biofilm at concentrations of 252 μg/ml compared to 532 μg/ml for gentamicin. This represents a 32- and 17-fold increase, respectively, in concentration required to eradicate the biofilm in the wound model compared to the MBEC, which is the concentration required to clear biofilm growing on a peg in a much simpler *in vitro* system (Table 1) (29).

Consistent with data for P. aeruginosa, an increased concentration of antibiotics was required to eradicate the MDRSA biofilm in the MBEC assay compared to planktonic cells as determined with the MIC. However, vancomycin combined with gentamicin was effective in decreasing the concentrations of antibiotics required to inhibit growth in both the MIC and the MBEC assays. Previous clinical use of these antibiotics involved the combination of 1 g vancomycin and 240 mg gentamicin with 10 ml calcium sulfate (i.e., 46.3 mg of vancomycin and 11.1 mg gentamicin per void). These levels of antibiotics did not eradicate the MDRSA biofilm in this model, although exposure to 100% loaded beads for 7 days did cause a 0.8-log reduction in viable counts, suggesting extended exposure time results in greater efficacy against biofilm (30).

Together, our data indicate that the biofilm in the collagen wound model has decreased susceptibility to antimicrobials, a hallmark of the biofilm mode of growth. Conditions are challenging for the microbes because the combination of the biofilm mode of growth and the collagen matrix may limit oxygen availability to the biofilm; furthermore, SWF is a nutrient-limited medium. These factors may contribute to more robust biofilm formation than that in an MBEC assay, potentially more like that found in a DFI. Of course, there are additional factors which may occur in a DFI, for example, the presence of host immune cells, physiological factors such as hypoxia, the presence or absence of exudate, and wound drainage. We established our biofilm over 72 h. It is difficult to say how long a biofilm takes to form *in vivo*, but it is likely to occur over a sustained period of time, and the maturation stage of the biofilm is likely to affect its susceptibility to antibiotics. This model is limited in that it is a closed system and therefore antibiotics will accumulate, whereas in wounds exudate is likely to drain or be absorbed by wound dressings. However, there is very little available pharmacokinetic data for antibiotics in DFU for the antibiotics used in this study, and because of poor vasculature in DFU, wound draining may be less critical. Furthermore, the stability of antibiotics *in vivo* may be different from that of this *in vitro* model. Nonetheless, it seems likely our model offers a much more relevant environment than agar plate zone inhibition assay, poly(methyl-methacrylate) (PMMA), or cellulose approaches to modeling biofilm in a wound-like context because it enables the concentrations and efficacy of antibiotics in an environment rich in collagen and serum proteins.

We also found that the location of antibiotics released from the calcium sulfate beads was variable, depending on the antibiotic used. The interaction of antibiotics with collagen also varied. Gentamicin did not show any evidence of interaction with collagen in the wound model, consistent with data observed for collagen-derived gentamicin release devices (31). Tobramycin showed a small concentration gradient across the collagen wound model at 50% and 100% of the CRC and was also more readily released from the calcium sulfate beads, reflected in its greater efficacy in biofilm eradication than gentamicin. This is surprising because tobramycin and gentamicin have similar chemical structures; therefore, this finding warrants further investigation. Gentamicin showed increased interaction with collagen in the wound model when combined with vancomycin as well as increased release from the calcium sulfate beads. Vancomycin does show some albumin and collagen binding activity and therefore might influence the interaction of gentamicin with the wound model (32, 33). The combination of gentamicin and vancomycin has also shown increased elution profiles from bone cements (34), likely due to the increased pore size in the cement material caused by vancomycin, which is a much larger molecule than gentamicin.

Binding of antimicrobials to serum proteins is an important factor in antibiotic efficacy *in vivo*, and the high level of FBS in the medium was introduced to mimic this. Similarly, type 1 collagen, which is the substrate for biofilm formation, is the predominant form of collagen in the dermis; therefore, the interaction of the biofilm and antimicrobials in this model may be closer to the *in vivo* situation than other models, such as those that use cellulose or PMMA discs as substrates to allow biofilm formation. The biofilm is also allowed to mature for 3 days where most biofilm models utilize biofilms established over 24 h, although *in vivo* wounds are considered nonhealing after 2 months, suggesting that biofilms mature over weeks rather than days in DFI.

Previous elution studies of antibiotics from calcium sulfate beads combined with daptomycin, moxifloxacin, fusidic acid, vancomycin, gentamicin, tobramycin, and rifampin showed sustained release of high concentrations of these antibiotics for a minimum of 14 days, stretching to 42 days (35–37). In the current model, between 49% and 83% of antibiotics loaded in the beads were detected in the collagen phase or bathing media, suggesting that the rest of the antibiotic was still located in the beads. Therefore, we did observe sustained release of antibiotics from the beads substantially above the MIC over 3 and 7 days, which may be why antibiotics showed such high efficacy against the susceptible P. aeruginosa strain in our model at the clinically available concentration.

Studies with several types of antibiotics present in calcium sulfate beads have been conducted and consistently show that therapeutic concentrations of antibiotics are achieved in the tissue of the DFI (38–45), although this is not the case for every patient (46). For example, one study did report significantly reduced vancomycin tissue concentrations in diabetics compared to nondiabetics after cardiac surgery (47). Therapeutic levels of antibiotics constitute concentrations in the tissue above the MIC for invading microbes. Bacterial infections are likely to be in the biofilm mode of growth in DFIs, so they would have an increased MIC. Therefore, standard therapeutic concentrations of antibiotics may not be relevant in these infections. These factors may contribute to the high rate of nonhealing ulcers and surgical intervention in DFI (6, 48–50).

Prolonged antibiotic use can also render patients more susceptible to infections such as Clostridium difficile (51). The gut microbiota is thought to be important in immunity and inflammation as well as in insulin sensitivity, and therefore the use of systemic antibiotics can have an adverse effect (52, 53). In these instances, packing a wound with antibiotic-loaded beads may avoid problems caused by systemic antibiotics while allowing close interaction between the antibiotics and the biofilm and release of high antibiotic concentrations. This route of administration may avoid systemic toxicity and drug interactions as well as decrease the risk of antimicrobial resistance induction by achieving high concentrations at the site of infection (54, 55). High aminoglycoside concentrations have been associated with instances of neuromuscular blockade, although this effect was mitigated by calcium (56), and there is little evidence from extensively used gentamicin-containing implants to suggest that this is a clinically relevant problem.

Data on the clinical efficacy of various wound dressings and topical antimicrobials is insufficient to allow clinicians to make informed decisions on best practices (50, 55, 57, 58), and treatment of infected DFUs is variable between different National Health Service settings (1). Few studies have been done to assess the effect of local antibiotics in DFI and ultimately wound closure (59); however, some studies have shown promising results with an antimicrobial peptide, pexiganan, as a cream (55) and with gentamicin incorporated into a collagen implant (60), as well as with tobramycin-impregnated calcium sulfate pellets according to a case study (61). Elution of vancomycin from calcium sulfate beads compares well with widely used PMMA bone cement that can also be loaded with vancomycin, tobramycin, or vancomycin in combination with tobramycin and formed as beads (30, 62), although unlike calcium sulfate, PMMA is not absorbable *in vivo* and therefore requires further intervention for removal.

In conclusion, the collagen wound model developed in this study has permitted the movement of antimicrobials through a physiologically relevant substrate, allowing a mature biofilm to be tracked and the resultant effect on the biofilm to be measured. Our *in vitro* data suggest that antibiotics combined with calcium sulfate beads have potential in the treatment of nonhealing DFI, as they provide sustained release of antibiotics well above the MIC while potentially avoiding systemic toxicity associated with prolonged systemic antibiotic use. The tissue concentrations reached appear in our system to be sufficiently high to be effective against mature biofilm and to even significantly reduce viable counts for a multidrug-resistant biofilm.
